# Impact of Sports Activity on Medium-Term Clinical and Radiological Outcome after Reverse Shoulder Arthroplasty in Cuff Deficient Arthropathy; An Institutional Register-Based Analysis

**DOI:** 10.3390/jcm10040828

**Published:** 2021-02-18

**Authors:** David Endell, Laurent Audigé, Alexandra Grob, Hans-Kaspar Schwyzer, Michael Glanzmann, Alex Marzel, Markus Scheibel

**Affiliations:** 1 Department Shoulder and Elbow Surgery, Schulthess Clinic, 8008 Zurich, Switzerland; david.endell@kws.ch (D.E.); grob.alexandra@gmx.ch (A.G.); hans-kaspar.schwyzer@kws.ch (H.-K.S.); michael.glanzmann@kws.ch (M.G.); 2 Teaching, Research and Development Department, Schulthess Clinic, 8008 Zurich, Switzerland; Laurent.Audige@kws.ch (L.A.); Alex.Marzel@kws.ch (A.M.); 3 Center for Musculoskeletal Surgery, Charité-Universitaetsmedizin, 10117 Berlin, Germany

**Keywords:** shoulder surgery, reverse shoulder arthroplasty, sports, radiological outcomes

## Abstract

There is a lack of consensus on what physicians can recommend and what patients can expect concerning sports activity after reverse shoulder arthroplasty (RSA). The purpose of this retrospective register-based observational study was to investigate the association between participation in sports or physical activity involving the upper extremity and 5-year clinical and radiological outcomes for primary RSA patients. We screened the institutional arthroplasty registry for patients reporting the type and level of sports postoperatively after primary, unilateral RSA due to rotator cuff deficiency. One hundred thirty-eight patients with clinical and radiological outcomes documented at a minimum 5-year follow-up were divided into three groups comprising those who participated regularly in: sports mainly involving the upper extremity (sports upper extremities, SUE, *n* = 49), sports mainly involving the lower extremities (sports lower extremities, SLE, *n* = 21), and those who did not participate in sports at all (no sports, NS, *n* = 68). The participants had a mean age of 72 years (standard deviation (SD) 8) and were overall predominantly female patients (62%). Primary clinical outcomes included the Constant Score (CS) and Shoulder Pain and Disability Index (SPADI). Secondary radiographs were analyzed for radiolucent lines (RLL), signs of glenoid or humeral prosthesis loosening, bone resorption, bone formation, and scapular notching. A total number of 8 senior surgeons were involved in treatment of patients, and two types of prosthesis were used. The SUE group had non-significantly higher mean scores for CS (75 points) and SPADI (88 points) compared to SLE (71 and 78 points, respectively) and NS patients (66 and 78 points, respectively) (*p* ≥ 0.286). The incidence of RLL around the humeral diaphysis was higher in NS compared to SUE patients (32% versus 12%, respectively) (*p* = 0.025); all other radiological parameters were similar between the groups. There were no cases of loosening in the SUE group that led to revision surgery. Patients engaging in sports activities involving the upper extremity show similarly good functional scores 5 years post-RSA as the other groups, without additional signs of implant loosening as a result of increased shoulder use.

## 1. Introduction

With the rate of reverse shoulder arthroplasty (RSA) continuously rising worldwide, it has acquired the status as the most frequently implanted endoprosthesis in the shoulder joint in the last decade [1,2,3]. Reverse shoulder arthroplasty has shown good clinical long-term results [4], which has led to surgeons gaining more confidence and extending the indications to more active patients. Recent evidence has shown that patient expectations and demands after RSA are high, particularly with regard to the effects surgery and even the implant type may have on postoperative participation in sports [5,6,7].

While clinical and radiological outcome parameters are well established and show good results after implantation [4], there is scant scientific evidence on the outcome of patients participating in sports after RSA. Two systematic reviews and meta-analysis have identified several scientific approaches made to investigate sports related outcomes after shoulder arthroplasty, yet there is a vast heterogeneity of the methodological approaches and the implants used (anatomical total shoulder arthroplasty, hemi shoulder arthroplasty, RSA), and only few medium-term outcomes with 5 years have been reported [8,9]. The frequency and type of sport activities undertaken by RSA patients show that return to sport participation rates are as high as 74.9–85.5% after surgery [6,7,8,9]. Yet there is little knowledge about clinical and radiological outcomes, and there is a particular lack of a comparative cohort for those actively participating in sports mainly involving the upper extremities. Only one study observed good clinical function for 41 RSA patients who regularly participated in various low and high impact sports postoperatively without any signs of loosening at a mean time of 43 months [10]. Nonetheless, there is still no consensus on what patients can expect, particularly regarding radiological changes as a possible indicator of the associated risk of a consecutive painful implant loosening and what level of sports activity is recommended to prevent this adverse event after primary RSA.

The objective of this study was to investigate the relationship between participation in sports involving the upper extremity after RSA and the clinical performance of patients and, furthermore, the occurrence of radiographic signs of implant wear, bone resorption, or loosening at a follow-up of 5 years. We hypothesized that the clinical outcome of patients actively participating in sports is good, and that there is no significant association between a higher active demand of the shoulder and greater radiographic signs of implant deterioration.

## 2. Materials and Methods

### 2.1. Patient Selection

**Figure 1:**
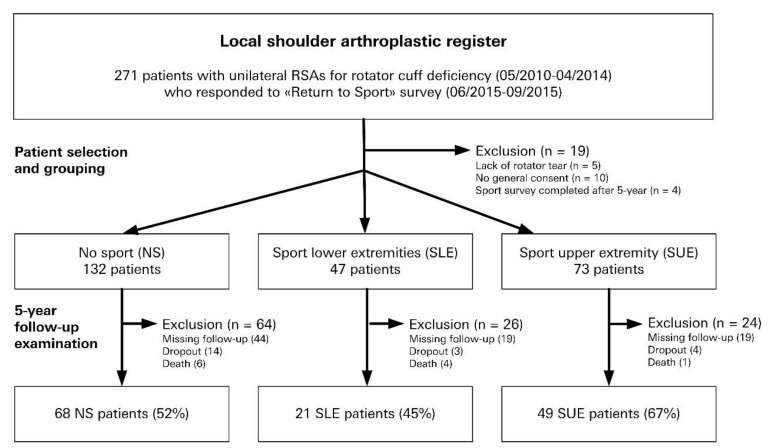
Flowchart visualization of the process of patient selection. RSA = reverse shoulder arthroplasty.

From a previous investigation [6], we identified patients who had undergone unilateral primary RSA between May 2010 and April 2014 and had responded to a postal survey about their level of sports participation (Figure 1). These patients were included in our analysis if they had completed a clinical and radiological follow-up examination at 5 years, which was documented in a local shoulder arthroplasty register [11]. Any patient who did not have a rotator cuff tear as well as those declining consent for their clinical data to be used for research purposes were excluded. Three patient groups were designated according to the main type of sports undertaken after RSA and comprised: (1) patients who regularly participated in sports mainly focused on using the upper extremities (SUE), (2) patients who participated in sports mainly involving the lower extremities (SLE), and (3) patients who did not participate in any sporting activities at all (NS). 

The physical status of all patients was assessed preoperatively and documented according to the physical status classification system of the American Society of Anesthesiologists (ASA).

### 2.2. Surgery and Postoperative Rehabilitation Protocol

All surgical interventions were performed using a standardized deltopectoral approach by eight senior consultants specialized in shoulder surgery. After detachment of the subscapularis, each prosthesis was implanted according to the manufacturer’s instructions in a standardized position with 10–15° of humeral retrotorsion. The utilized RSA implants included the PROMOS Revers™ (Smith+Nephew, Hertfordshire, UK) and Univers Revers™ (Arthrex GmbH, Munich, Germany), with either 155° or 135° humeral inclination. The subscapularis tendon was reattached whenever reconstruction, which was dependent on tendon and muscle quality, was possible.

All patients followed a standardized postoperative rehabilitation protocol. During the first postoperative week, the shoulder was immobilized in a sling in internal rotation to control the level of pain, although early mobilization within the patient’s field of vision was permitted. For the first two weeks, post-RSA, only passive movements involving limited internal and external rotation were allowed followed by increasing the range of motion from the third postoperative week. Active mobilization was prescribed from the fifth postoperative week, and the length of the rehabilitation program differed based on each patient and their individual progress in shoulder function. At the 3-month postoperative time point, patients were permitted to resume all physical activities of daily living including non-contact sports only.

### 2.3. Radiological Assessment

Standardized radiographic images taken in internal/external rotation and axillary views at the 5-year postoperative follow-up were evaluated for the appearance of radiolucent lines (RLL) around the implant, signs of humeral or glenoid component loosening, bone resorption (including scapular notching), bone formation, and signs of implant wear, according to an international standard core set of radiographic parameters for shoulder arthroplasty monitoring [12]. Scapular notching was graded according to Sirveaux et al. [13]: notch limited to the scapular pillar (Grade 1), notch reaching the inferior screw of the base plate (Grade 2), and notch reaching above the inferior screw of the base plate (Grade 3). Occurrences of heterotopic ossifications around the implant were graded according to the modified Brooker classification [14]: islands of bone within the soft tissues around the shoulder (Grade 1), bone spurs from the proximal humerus or scapula, leaving at least one centimeter between opposing bone surfaces (Grade 2), and bone spurs from the proximal humerus or scapula, reducing the space between opposing bone surfaces to less than one centimeter (Grade 3). The appearance of RLL was graded based on an adapted method originally described by the working groups of Sperling [15] and Schoch [16] and categorized as either incomplete (Grade 1) or completely surrounding the implant (Grade 2). The width of RLL was also documented and classified as Grade 1a and 2a when smaller than 1.5 mm and Grade 1b and 2b for RLL widths greater than 1.5 mm. The evaluation process was performed independently by two trained orthopedic clinicians who were blinded to the information on the patients’ sports activities, and any discrepancies were resolved by consensus.

### 2.4. Clinical Follow-Up

Patients were examined at 6, 12, 24, and 60 months after surgery. Clinical parameters included: shoulder active range of motion (ROM) in flexion, abduction, external rotation in 0° abduction, and internal rotation according to the Apley Scratch Test, during which the patient attempts to touch the opposite scapula; shoulder strength in 90° abduction was determined using a spring balance (Pesola AG, Schindellegi, Switzerland), and functional outcome were based on the Constant Score (CS) [17,18] and patient-reported Shoulder Pain and Disability Index (SPADI) [19]. The occurrence of revision surgery was also recorded.

### 2.5. Data Management and Statistical Analysis

Register data were managed using the REDCap electronic data capture system [20] and exported for statistical analysis into Intercooled Stata version 14 (StataCorp LP, College Station, TX, USA). The types of sports practiced by RSA patients assigned to the SUE and SLE groups were described. Baseline patient demographics and shoulder status were tabulated separately per group using standard descriptive statistics and compared using statistical and clinical judgement to assess any differences that could influence the radiological and functional outcomes.

Radiological parameters were tabulated between groups using absolute and relative frequencies and compared using logistic regression analyses adjusted for sex and ASA classification. The outcomes of ROM, shoulder strength, CS, and SPADI were tabulated and graphically presented per group at baseline as well as at each follow-up. Comparative analyses for these parameters were conducted using generalized linear mixed models to account for repeated measurements at each follow-up, as required. Group differences at the 5-year follow-up were investigated using linear regression. The parameters of scapular notching, heterotopic ossifications, and internal rotation were compared using ordered logistic regression analyses. For all models, we included the respective baseline functional parameters and the baseline parameters of sex and ASA classification. Adjusted group outcome differences and 95% confidence intervals were calculated for the 5-year follow-up. All analyses were considered explorative, and statistical significance was set at 0.05.

### 2.6. Patient Selection and Group Matching

Of 271 unilateral primary RSA patients who provided information on their return to and participation in sports after RSA, 10 patients did not provide their informed consent and five did not have a rotator cuff tear (Figure 1). Another four patients had already completed the 5-year follow-up prior to completing the sports participation survey, 11 had died, 21 patients dropped out before 5 years post-RSA, and a further 82 were excluded due to missing 5-year follow-up data. Our final analysis database included 68 NS patients, 21 SLE, and 49 SUE, respectively.

Initially, patients who were actively and regularly participating in sports postoperatively were identified and their three main sports, and sports that were practiced alongside, were analyzed. While swimming, gymnastics, golf, tennis, bowling, and sailing were all categorized as sports producing a higher load and force coupling on the upper extremities, patients in the SUE group also commonly participated in other sports that were mainly practiced by SLE patients (Table 1). It is to be noted that while some sports, such as strength training, aquafitness, cross country skiing, and skiing, can in an adaption also be performed with a lower impact on the upper extremities, gymnastics does demand an unequivocal participation of the arms and shoulders. In the SUE group, only sports with an explicit higher demand of the upper extremities where included. All patients who were actively participating in sports postoperatively had been active prior to RSA; only 21% of NS patients had practiced sport before surgery.

The mean patient age at the time of surgery ranged from 71–72 years, with a similar distribution between the patient groups (Table 2). There was a greater proportion of female patients in the SUE and NS groups. Surgery was mostly performed on the dominant side. While half of the patients in the NS and SLE groups had an ASA class III status, this proportion was lower (37%) in the SUE group. 

## 3. Results

### 3.1. Radiological Evaluation

The most prevalent observations made from the 5-year follow-up radiographs included the presence of RLL around the implant and bone resorption and/or formation (Table 3). There were no signs of implant migration, shoulder joint displacement, wear of the implant articular surfaces, or implant breakage or disassembly.

Within the 5-year follow-up period, three patients had sustained fractures around the implant of which one involved the humerus and two the acromion. One SUE patient sustained a periprosthetic humeral shaft fracture during a non-sports related fall at home two weeks after RSA. The fracture was initially fixed with combined cerclage stabilization and plate osteosynthesis including allograft augmentation, but required revision with additional posterior plate fixation, which was performed within 3 weeks. Shoulder function was satisfactory for carrying out activities of daily living. However, mobility of the affected arm was limited to horizontal abduction and no overhead sports were possible, yet the patient did participate in swimming.

Another SUE patient sustained a periprosthetic acromion fracture type II according to Levy et al. [23] during the rehabilitation period at 2 months post-surgery, which was treated conservatively. The fracture healed and was described as stable at the 5-year follow-up; the patient reported good subjective shoulder function while golfing and reached a CS of 72.

From the NS group, the third patient sustained a periprosthetic acromion type III fracture according to Levy during a fall at 32 months after RSA. After initial conservative treatment failed, pseudarthrosis revision was performed via allograft interposition and double-plate fixation. Postoperative subjective shoulder function for this non-sport participant was satisfactory for activities of daily living.

At 5 years, three other relevant radiographical observations were made: an SLE patient and an NS patient were each identified with a zone of bone resorption around the reinsertion site of a latissimus dorsi transfer concomitant with the RSA; further treatment was not necessary because there was no evidence of humeral stem loosening. Additionally, the disruption of an osteosynthesis screw placed for fixation of an os acromiale 6 months prior to RSA surgery was observed in another NS patient; this patient did not participate in sport and reported intermittent minor impingement symptoms at follow-up.

**Figure 2:**
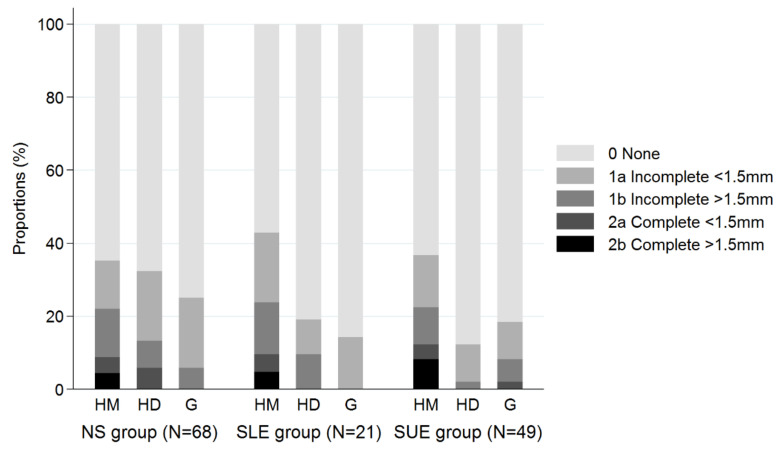
Radiological observation of radiolucent lines (RLL) appearance 5 years after RSA implantation. HM = Humeral metaphysis; HD = Humeral diaphysis; G = Glenoid.

Radiolucent lines were observed in almost half of all patients from the SUE and SLE groups (*p* = 0.953) (Table 3). Most RLL had a minimum thickness of 1.5 mm or less and were incompletely surrounding the implant (Figure 2). Grade 1b and 2a RLL were seen at the humeral diaphysis in one (2%) SUE patient, two (10%) SLE patients, and nine (13%) patients from the NS group (*p* = 0.053). On the other hand, Grade 2b RLL at the humeral diaphysis were not reported in any group. The NS group had the highest incidence of RLL at the humeral diaphysis (32%) followed by the SLE (20%) and SUE groups (12%) (*p* = 0.053), and was significantly higher compared to the SUE group only (*p* = 0.025). At the humeral metaphysis, overall complete RLL and incomplete RLL ≥ 1.5 mm (Grade 1b–2b) were observed in a very similar proportion of patients (21–24%) in all three groups (*p* = 0.984). Complete appearance of metaphyseal RLL over 1.5mm (Grade 2b) was observed in 3 (4%) NS patients, 1 (5%) SLE patient, and 4 (8%) SUE patients. At the glenoid side, between 14% and 25% of patients showed signs of RLL between the groups (*p* = 0.641). No incidence of complete RLL ≥ 1.5 mm at the glenoid was observed in any group. 

No cases of loosening and no cases of loosening associated revision surgery were documented in the SUE, SLE, or NS group.

Overall, there were signs of either bone resorption or bone formation on either the glenoid or humeral side in a range of 81–84% of patients from the three groups (*p* = 0.980). Scapular notching, particularly Grade 1 and 2, was commonly reported at the glenoid region (Table 4). Similar proportions were seen between the groups, although the NS group tended to have the highest rate of notching of 51% (*p* = 0.200). Heterotopic ossification was identified in 26 (38%) NS patients, 8 (38%) SLE patients, and 12 (24%) SUE patients with comparable levels of severity (*p* = 0.324) (Table 4).

### 3.2. Clinical Evaluation

**Figure 3:**
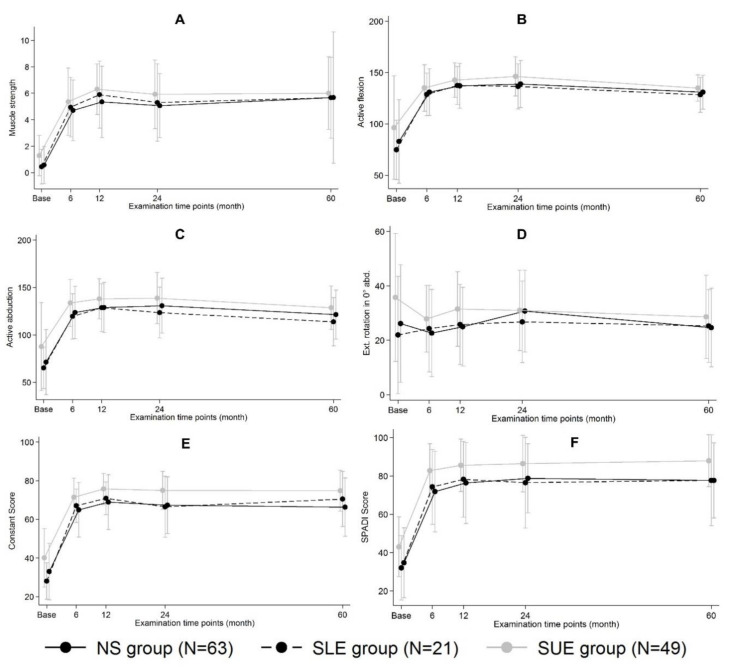
Development of clinical outcomes ((**A**) muscle strength; (**B**) active flexion; (**C**) active abduction; (**D**) external rotation in 0° abduction; (**E**) Constant Score; (**F**) SPADI Score) from baseline to 6, 12, 24, and 60 months after RSA implantation.

The observed outcomes of the clinical scores post-RSA increased comparably between the groups. Patients from the SUE group were observed to have non-significantly higher clinical performance scores already before and after surgery, as indicated by higher mean values of the CS (74.8 points; *p* = 0.286) and SPADI (88 points, *p* = 0.906) at 5 years. Figure 3 compares the trends in CS and SPADI scores at the 12-, 24-, and 60-month follow-ups and shows stable clinical outcomes for the SUE group. Postoperative assessment of active ROM indicated increased flexion and abduction in all groups, where SUE patients had the highest mean values of active flexion, abduction, and external rotation at 0° abduction compared to the SLE and NS groups (*p* ≥ 0.277) (Figure 3). After adjusting for the respective baseline values, sex, and ASA classification, active internal rotation was significantly greater for SUE patients (*p* ≤ 0.001) (Suppl. 1). Although SUE patients had a higher mean value of 6.1 kg for muscle strength in 90° abduction, this was not significantly different from mean strength reported for SLE and NS patients (*p* = 0.767).

## 4. Discussion

Five years after RSA, patients who resumed sporting activities mainly involving the upper extremity showed similar good shoulder function in comparison to NS and SLE. Besides better outcomes for internal rotation, other reported outcomes, such as increased ROM and higher strength as well as the clinical- and patient-reported outcomes of CS and SPADI, showed no significant difference compared to NS and SLE, respectively. Although no difference in general appearance of RLLs was observed between the groups, the SUE cohort had a lower incidence of RLL at the humeral diaphysis compared to those who did not undertake any sports before or after RSA.

One would expect that implant wear and the rate of irregularities at the bone-implant surface to be higher in patients who apply repetitive cycles of force and load on their upper extremities while participating in sports. We did not find any tendencies suggesting increased prosthesis wear or stem loosening in the SUE collective at a medium-term postoperative follow-up of 5 years. When comparing the outcome of RLL, these lines appeared to be significantly lower in SUE patients compared to those who did not participate in sports at all; the etiology of RLL in our NS patients cannot be speculated. Simovitch et al. [10] investigated a patient collective of similar age to our study, who had a mixed level of sports participation and were followed up after a mean time of 43 months post-RSA. Here, radiolucent lines were observed in 17% of humeral stems and localized only in one zone; the presence of any grade of RLL in our study patients (SUE) was notably higher (49%). But Simovitch et al.’s investigation lacked a classification of the RLL identified, which makes any comparison to our results difficult. As with our study patients, Simovitch et al. [10] also reported no cases of humeral stem loosening at final follow-up. Notching was observed in fewer patients (7%) [10] compared to our study; this may be explained by our longer minimum follow-up interval of 5 years for all patients, as Lévigne et al. describe a progressiveness in frequency of appearance of scapular notching in their cohort with a mean follow-up time of 51 months (range, 24–206 months) [24]. Our SUE and NS patients shared similar rates for scapular notching (45% and 51% respectively), which were slightly lower than the rate of notching (62%) in a non-sports related study with a mean follow-up of 47 months [24].

The postoperative clinical outcomes reported for SUE patients in this study indicate their excellent clinical performance after 5 years. One must consider a potential preselection bias associated with the generally better preoperative clinical performance of SUE patients, as indicated by the higher mean baseline CS and SPADI scores. Due to their preoperative participation in upper extremity sports, SUE patients may have a better pre-existing constitution of the shoulder muscles and joint. In this study, SUE patients do not obtain higher changes of the observed clinical scores after RSA surgery than patients from NS and SLE, but appear to obtain higher medium-term functional results, although these results are non-significant.

Only a few systematic reviews or meta-analyses have considered the return to sport after RSA [8,9]. From these reports, 74.9% [8] and 77% [9] of patients who performed sports preoperatively returned to sports after RSA. A similar proportion of 77% was reported for the RSA population from which our analysis groups were selected [6]. The most common sports with the highest return rates were swimming, hiking, fitness/gymnastics, and cycling, and these activities were also most common for the SUE patients in this study. Generally, patients from the SUE group tended to participate in a variety of sports in addition to the sport they most often practiced. The sport activities most commonly practiced in the SLE and SUE group appear to be non-contact sports. This is in line with the recommendations of American Shoulder and Elbow Society members who were surveyed about their advice concerning sports participation after RSA [25]; Golant et al. found that the majority of surgeons recommended low-impact sports after RSA, although they were more hesitant about allowing patients to undertake sports after RSA compared to anatomical shoulder arthroplasty design.

This study has several limitations. This is an observational study with potential systematic baseline differences between the groups, notably regarding the overall shoulder fitness of the included patients, as indicated by higher preoperative CS and SPADI in SUE, although non-significant. While statistical adjustment was applied to account for some observed differences, some residual confounding cannot be excluded. There is a relatively low number of patients in each group and, thus, the detection of any relevant changes in rare rates at a medium-term follow-up is likely underpowered. Generally, a longer follow-up, ideally over 10 years, is needed to evaluate the long-term survival rates of RSA in sports-participating patients. In a setting of patients with a mean age of 71 years and older at time of surgery, the inability to appear at a 5 year follow-up assessment is not uncommon, and high rates of follow-up are difficult to achieve. Additionally, is it theoretically possible that patients showing loosening or requiring revision surgery elsewhere could have been missed. The patient follow-up limited by older age and lower general health, as documented by the ASA classification, was more notably in patients in the SLE and NS groups. Patients who tended to use their implants most actively in sports (SUE), are also those who tended to be in better general health, as indicated by the ASA classification, and could therefore be more likely to appear at the follow-up assessment. The methodology of a registry-based study includes the possible confounding factor of multiple surgeons being involved; a strict homogeneity in surgical technique and evaluation can only be controlled to a certain extend. 

## 5. Conclusions

The observed changes in clinical scores and the occurrences of abnormal radiological outcomes in patients actively performing sports of the upper extremities 5 years post-RSA were similar to patients mainly participating sports of the lower extremities and those not performing any sports.

## Figures and Tables

**Table 1:** Type of sports practiced between return-to-sport groups SLE and SUE.

	SLE Group (*n* = 21)		SUE Group (*n* = 49)	
	*n*	%	*n*	%
**Sports involving the upper extremities**				
Swimming	-		33	67%
Gymnastics	-		22	45%
Golf	-		6	12%
Tennis	-		6	12%
Bowling	-		3	6%
Sailing	-		1	2%
**Sports that may not Involve the Upper Extremity**				
Hiking	10	48%	29	59%
Cycling	7	33%	20	41%
Strength training	6	29%	11	22%
Walking	4	19%	10	20%
Aquafitness	3	14%	6	12%
Jogging	2	10%	2	4%
Horse riding	2	10%	2	4%
Skiing	2	10%	7	14%
Gymnastics for senior	1	5%	0	0%
Soccer	1	5%	1	2%
Cross-country skiing	1	5%	2	4%

**Table 2:** Baseline patient demographics and shoulder status.

	NS Group		SLE Group		SUE Group		SdtDiff	
	*n*	Mean(SD)	*n*	Mean(SD)	*n*	Mean(SD)	SLE vs. SUE	NS vs. SUE
Age at surgery	68	72 (7)	21	71 (8)	49	72 (8)	0.140	0.008
Gender (*n*,%)							0.137	0.145
Female	45 (66)		11 (52)		29 (59)			
Male	23 (34)		10 (48)		20 (41)			
ASA Classification (*n*,%)							0.291	0.300
I	1 (1)		-		1 (2)			
II	32 (47)		11 (52)		30 (61)			
III	35 (51)		10 (48)		18 (37)			
Dominant Side Operated (*n*,%)							0.084	0.026
Non-dominant	16 (24)		4 (19)		11 (22)			
Dominant	52 (76)		17 (81)		38 (78)			
Indication for RSA (*n*,%)							0.272	0.284
RC tear without Arthrosis	10 (15)		3 (14)		3 (6)			
RC tear with Arthrosis	58 (85)		18 (86)		46 (94)			
Prothesis type (*n*,%)							0.239	0.105
PROMOS Revers™ ^†^	41 (60)		14 (67)		27 (55)			
Univers Revers™ ^‡^	27 (40)		7 (33)		22 (45)			
SPADI Score	68	35 (18)	21	32 (17)	49	43 (16)	0.674	0.489
Constant Score	63	33 (15)	21	28 (9)	49	40 (15)	0.957	0.474

SdtDiff = standardized difference calculated as the absolute difference between group means divided by the common standard deviation [21], where values closest to 0.10 indicate stronger group similarity; ASA = American Society of Anesthesiologists; SD = standard deviation; RC = Rotator cuff; ^†^ Smith+Nephew, Hertfordshire, UK; ^‡^ Arthrex GmbH, Munich, Germany

**Table 3:** Radiological parameters 5 years after RSA implantation.

	NS (*n* = 68)	SLE (*n* = 21)	SUE (*n* = 49)	
Core set parameter groups ^†^	*n* (%)	*n* (%)	*n* (%)	*p*-value ^‡^
1- Implant migration	-	-	-	
2- Radiolucency	33 (49)	10 (48)	24 (49)	0.953
3- Signs of Shoulder Joint Displacement	-	-	-	
4- Bone Resorption/Bone Formation	55 (81)	17 (81)	41 (84)	0.980
5- Wear of The Implant Articular Surfaces	-	-	-	
6- Fractures Around The Implant	1 (1)	-	2 (4)	*n*.*p*.
7- Implant Breakage/Disassembly	-	-	-	
8-Other Relevant Observation	2 (3)	1 (5)	-	*n*.*p*.

^†^ according to an international consensus standard [22]; ^‡^ *p*-value = adjusted binomial regression analyses adjusted for gender, ASA classification; n.p. = regression analysis was not performed due to the limited number of events observed.

**Table 4:** Radiological observation of bone resorption and bone formation 5 years after RSA implantation.

	NS (*n* = 68)	SLE (*n* = 21)	SUE (*n* = 49)	
Bone resorption/Bone formation parameters ^†^	*n* (%)	*n* (%)	*n* (%)	*p*-value ^‡^
**Bone resorption**				
Humeral metaphysis (above the surgical neck)				0.837
No	65 (96)	20 (95)	44 (90)	
Calcar region	3 (4)	-	3 (6)	
Calcar region and Tuberosities	-	1 (5)	-	
Tuberosities	-	-	2 (4)	
Humeral diaphysis				0.458
No	53 (78)	15 (71)	40 (82)	
Yes	15 (22)	6 (29)	9 (18)	
Scapular notching grade Nerot-Sirveaux [13] classification				0.200
No sign of notching	33 (49)	15 (71)	27 (55)	
Grade 1	24 (35)	5 (24)	18 (37)	
Grade 2	11 (16)	1 (5)	4 (8)	
**Bone formation/ossification**				
Humeral metaphysis (above the surgical neck)				0.745
No	51 (75)	14 (67)	39 (80)	
Heterotopic	14 (21)	5 (24)	7 (14)	
Orthotopic	1 (1)	1 (5)	3 (6)	
Orthotopic and Heterotopic	2 (3)	1 (5)	-	
Scapula				0.745
No	36 (53)	8 (38)	23 (47)	
Heterotopic	18 (26)	6 (29)	9 (18)	
Orthotopic	14 (21)	7 (33)	17 (35)	
Modified Brooker [14] classification				0.324
No bone formation	42 (62)	13 (62)	37 (76)	
Grade 1	15 (22)	4 (19)	7 (14)	
Grade 2	10 (15)	4 (19)	5 (10)	
Grade 3	1 (1)	-	-	

^†^ according to an international consensus standard [22]; ^‡^ *p*-value = adjusted logistic regression analyses adjusted for gender, ASA classification; parameters were dichotomized (presence/absence) for this comparison to the limited number of events observed. Scapular notching and Modified Brooker classification were compared using ordered logistic regression analyses without dichotomization.

## Data Availability

The Schulthess Shoulder Arthroplasty Registry data availability policy and procedures are outlined in Marzel et al. [11].
